# Depressive Symptoms and Cognitive Function in Older Adults: A Cross-Lagged Network Analysis

**DOI:** 10.1155/2024/6166775

**Published:** 2024-09-21

**Authors:** He-Li Sun, Pan Chen, Wei Bai, Qinge Zhang, Sha Sha, Zhaohui Su, Teris Cheung, Gabor S. Ungvari, Todd Jackson, Yuan Feng, Yu-Tao Xiang

**Affiliations:** ^1^Unit of Psychiatry, Department of Public Health and Medicinal Administration and Institute of Translational Medicine, Faculty of Health Sciences, University of Macau, Macao SAR, China; ^2^Centre for Cognitive and Brain Sciences, University of Macau, Macao SAR, China; ^3^Department of Epidemiology and Biostatistics, School of Public Health, Jilin University, Changchun, China; ^4^Beijing Key Laboratory of Mental Disorders, National Clinical Research Center for Mental Disorders and National Center for Mental Disorders, Beijing Anding Hospital; Advanced Innovation Center for Human Brain Protection, Capital Medical University, Beijing, China; ^5^School of Public Health, Southeast University, Nanjing, China; ^6^School of Nursing, Hong Kong Polytechnic University, Hong Kong SAR, China; ^7^Section of Psychiatry, University of Notre Dame Australia, Fremantle, Australia; ^8^Division of Psychiatry, School of Medicine, University of Western Australia, Perth, Australia; ^9^Department of Psychology, University of Macau, Macao SAR, China

**Keywords:** cognitive decline, cross-lagged network analysis, depression, executive function

## Abstract

**Background:** Depressive symptoms commonly co-occur with cognitive decline in older adults. However, prospective interrelationships between different cognitive function domains and depressive symptoms are not well understood. This study evaluated prospective interrelationships between depressive symptoms and cognitive functioning components among individuals aged 50 years or older from a perspective of network analysis.

**Method:** Longitudinal data from the English Longitudinal Study of Aging were analyzed. Depressive symptoms were measured with the eight-item Center for Epidemiologic Studies Short–Depression Scale. Cognitive functions assessed included memory, orientation, and executive function. Contemporaneous network analyses were conducted using mixed graphical model, while a temporal network model was assessed using cross-lagged panel network model. To identify important predictors and outcomes, centrality indices, including expected influence, out-expected influence, and in-expected influence, were calculated.

**Results:** A total of 6,433 older adults were included in the network analysis. Baseline “Not enjoy life” (CESD-6) was negatively associated with executive function at the follow-up assessment. Moreover, improvements in “Everything was an effort” (CESD-2) and “Loneliness” (CESD-5) were related to less future decline of executive function and memory ability. Furthermore, analyses suggested targeting “Lack of happiness” (CESD-4) could be useful in reducing the co-occurrence of depression and cognitive decline among older adults.

**Conclusions:** This network analysis study highlighted dynamic interrelationships between depressive symptoms and cognitive decline in older adults. Findings suggest that interventions targeting specific depressive symptoms may have the potential to alleviate declines in executive function and memory for this population.

## 1. Introduction

As the world's population continues to age, the issue of cognitive impairment has become increasingly pressing [1]. Cognitive decline is an inevitable consequence of aging and can vary in severity from mild cognitive impairment to dementia. Based on recent estimates, around 15.56% (95% CI: 13.24%–18.03%) of people aged 50 and older suffer from mild cognitive impairment [2], while the prevalence of all-cause dementia is 6.97% (95% CI: 5.46%–8.64%) worldwide [3]. In light of such data, interventions aiming to alleviate cognitive decline in vulnerable older adults are of great importance.

Previous research has found that depressive symptoms can have a significant influence on both the onset and progression of cognitive decline [4–6]. To illustrate, a meta-analysis [7] indicated depressive symptoms were significant risk factors for the progression to dementia in individuals with mild cognitive impairment. Furthermore, a recent population-based cohort study indicated that older adults who experienced a remission of depressive symptoms did not exhibit accelerated cognitive decline in the follow-up period [8]. In contrast, participants with poorer cognitive performance often experience a higher risk for elevated depressive symptoms [9]. Moreover, several studies have found that associations between depression and cognition might be mediated through negative social experiences such as social isolation and marital conflict. For example, depression–related social isolation could limit normal social interactions and impede the development of cognitive capacities, ultimately impairing the maintenance of cognitive reserves [10, 11]. Researchers have also found that positive marital quality is associated with both a better mental health status and enhanced cognitive performance in older adults [12, 13].

Traditionally, studies have evaluated links between severity of cognitive declines and depression using total scores from standardized scales and exploring their associations at a syndrome/disorder level. Nonetheless, depression is associated with different cognitive components including processing speed, working memory, and executive functions [14, 15], so there may be utility in exploring potential relationships between different cognitive functions and specific depressive symptoms.

Network analysis is a novel approach to evaluating how individual components (symptoms) of a construct (e.g., a psychiatric disorder/syndrome) can influence other components (symptoms) within the same construct or other constructs [16]. Depending on the type of dataset, network analysis methods can be divided into two categories. The first category is contemporaneous network analysis which depicts associations between pairs of nodes assessed at a single point in time. Popular models used for contemporaneous networks include Gaussian graphical models (GGM), Ising models, and mixed graphical models (MGMs) [17]. The second category is temporal network analysis, which is used for longitudinal panel data to assess relationships between pairs of nodes over time via cross-lagged network models (CLPN) [18]. Through CLPN, for instance, researchers can determine whether depressive symptoms at baseline can predict depressive symptoms and cognitive function at follow-up assessments after controlling for covariates at baseline. Similarly, baseline cognitive function may predict subsequent depressive symptoms and/or cognition at follow-up. CLPN provides novel insights that could assist clinicians in diagnosing and managing the course of cognitive impairment or depressive symptoms in practice as well as guiding clinical trials that aim to improve cognitive impairment associated with depressive symptoms.

In recent years, several network analyses have explored interrelationships between different depressive symptoms and cognitive deficits [19–21]. However, most of these studies were based on cross-sectional datasets, leaving dynamic relationships between variables largely unexplored [19]. Moreover, although a few longitudinal studies have explored network changes between depression and cognition, they involved limited cognitive component such as the executive function domain [21], or focused on highly specific population subgroups such as midlife community-dwelling adult women [20].

In light of these limitations, the aim of this study was to assess interrelationships between multiple depressive symptoms and cognitive components (e.g., memory, orientation, and executive function) within a nationally representative population sample of older adults using both contemporaneous and temporal network models.

## 2. Methods

### 2.1. Participants and Study Design

This study was part of the English Longitudinal Study of Aging (ELSA) [22]. The ELSA is an ongoing observational, longitudinal project involving community-dwelling adults aged over 50 years living in England. The first wave of data collection began in 2002, and assessments have continued every 2 years thereafter. The ELSA survey covers multiple aspects of life among older adults, including demographics (i.e., age, gender, marital status, educational status, smoking, and drinking habits). In addition, the ELSA has collected information about individual physical and psychosocial health, cognitive function, work experience, and retirement. Our study was based on the most recent two waves of the ELSA before the COVID-19 pandemic onset: wave 8 (2016–2017) served as the baseline assessment and wave 9 (2018–2019) comprised the follow-up assessment. Participants were included if they (1) completed assessments of depression, memory, orientation, and executive function in wave 8 (2016–2017) and wave 9 (2018–2019) and (2) were aged 50 years or older at wave 8 (2016–2017). Ethical approval of the ELSA was granted from the National Research and Ethics Committee (MREC/01/2/91); all participants provided written informed consent.

### 2.2. Measurements

Depressive symptoms were assessed using the eight-item version of the CESD [23, 24]. The CESD is a widely used, highly reliable questionnaire for evaluating depression [25]. Each item is scored as either “0” (absence) or “1” (presence), and total scores range from 0 to 8, with higher scores indicating more severe depressive symptoms.

Following previous ELSA research [8], cognitive components including memory, orientation, and executive function were assessed in this study. The evaluation of memory involved two recall tests, each consisting of 10 unrelated words. Participants were asked to recall these words either immediately or after a short delay, with one point awarded for each correctly recalled word. Total memory scores ranged from 0 to 20 based on the sum of points across both tests. Orientation was assessed by asking participants to name the day, month, year, and day of the week, with one point allocated for each correct answer; total orientation scores ranged from 0 to 4. Executive function was assessed using a verbal fluency test, in which participants were instructed to list as many animal names as possible within 1 minute. Each correct response was assigned one point. To facilitate the interpretation of results, original scores were transformed into categorical variables based on the ELSA official standard (detailed category method was described in the supplemental methods), generating a total executive function index score that ranged from 0 to 9 to represent different levels of executive function. Higher values indicated better cognition function. All cognitive tests have established psychometric properties based on past work [26–28]. Additionally, basic demographic and clinical data (e.g., age, sex, education level, marital status, current smoking status, and alcohol consumption status) were collected.

### 2.3. Statistical Analysis

#### 2.3.1. Data Imputation and Redundant Identification

To reduce bias and increase the efficiency of calculating point parameter estimates, missing data on covariates were addressed using multiple imputation with the *mice* package [29] rather than casewise deletion. In this study, five imputed datasets were created, and each dataset was run for a sum of 1,000 iterations before conducting network analysis.

Redundant nodes, which refer to items that are highly correlated, were assessed as a critical initial step before constructing a network model [18, 30]. To test redundancy, we used a goldbricker procedure examining node pairs with similar connections to other nodes [31]. If a pair of nodes had more than 75% similar connections to other nodes, the pair of nodes were considered redundant and then removed or collapsed from the final network analysis [31]. The goldbricker procedure was performed with the *networktool* package (Jones and Jones, 2018).

#### 2.3.2. Contemporaneous Network Estimation

Given that the data for network analyses consisted of categorical, binary, and continuous variables, contemporaneous networks of depression and cognition were generated using MGM, which is applicable to mixed variable datasets [32]. In the MGM network model, items were visualized as nodes, while associations between items, after adjusting for the influence of other nodes and covariates, were visualized as edges. Thicker edges indicated stronger relationships between nodes. Green and red colors indicated positive and negative associations, respectively. To mitigate the risk of false-positive associations, we applied the least absolute shrinkage and selection operator (LASSO) method. The penalty strength of the LASSO is regulated by a tuning parameter *λ* [33], which was selected using the extended Bayesian information criterion (EBIC) [34]. MGM was carried out using the *mgm* package (Haslbeck and Waldorp, 2015).

To assess centrality within the contemporaneous network, we used two-step Bridge Expected Influence (BEI), which captures a node's overall connectivity [35]. A higher BEI value indicates that a node has a stronger influence on nodes from the other community. Furthermore, we calculated the percentage of predictability of a given node that could be explained by other nodes in the model [36].

#### 2.3.3. Temporal Network Estimation

The temporal network model was constructed using CLPN, which investigates dynamic relationships between symptoms over time [18]. In CLPN theory, nodes represent symptoms, and arrows between symptoms represent directions of the associations. Green arrows reflected positive associations, and red arrows indicated negative associations. The thickness of arrows indicated the strength of associations, with thicker arrows indicating stronger associations between nodes. CLPN was performed using the *glmnet* package [37].

The main step in constructing CLPN involves fitting a series of regularized regression models to estimate the autoregressive coefficients and cross-lagged coefficients across different assessments over time [18]. Autoregressive effects refer to the influence of a node on itself over time, where a symptom at a given time point predicts its status at a subsequent time point. Cross-lagged effects refer to the influence of a node at one time point on other nodes at a subsequent time point. Both types of effects reflect temporal relationships between nodes within a network model. Weak edge weights were shrunk to exactly zero using LASSO with 10-fold cross-validation. Age, sex, education level, marital status, current smoking status, and alcohol consumption status were controlled for as potential confounding factors [38–40].

Symptom centrality estimates for the temporal networks were evaluated using two types of Expected Influence (EI) values [34]. First, Out-EI reflects the degree to which a symptom could predict other symptoms at a subsequent time point. Second, In-EI is the degree to which a symptom is predicted by other symptoms at a subsequent time point, with larger values indicating greater centrality [41]. These centrality estimates may provide insight into the relative importance of each symptom in the network model and help identify key nodes that play a critical role in temporal dynamics of the network model.

#### 2.3.4. Network Stability and Accuracy

The stability of network models refers to the rank order of the centrality values, including BEI in the contemptuous network as well as Out-EI and In-EI in the temporal network. Stability can be estimated using a correlation stability (CS) coefficient via case-bootstrapping procedure [42]. In general, when a CS-coefficient is larger than 0.50, the network is considered to be prefectly stable. Network accuracy reflects the stability of edge weights and was evaluated based on a bootstrapped 95% confidence interval (CI) via a nonparameter bootstrapping procedure. If the bootstrapped 95% CI of an edge weight was narrow, the edge was regarded as reliable [42]. To test whether there were significant differences in centrality and edge weights, differences between values were computed and analyzed using appropriate statistical tests [42]. Stability and accuracy tests were evaluated using the *bootnet* package [43]. All analyses were performed using R program (4.0.1 Version) [44].

## 3. Results

### 3.1. Demographic Characteristics and Node Redundancy

The original sample size at the baseline was 8,445 participants; of these, 6,433 fulfilled the study entry criteria and were included in final analyses. Table S1 presents the demographic characteristics of the sample. The average age of participants was 68.47 years (standard deviation [SD] = 8.95 years); 43.5% were men and 65.5% were married. In the network redundancy test, the goldbricker procedure did not find redundancy between the nodes. The labels and item descriptions for each of the depressive symptoms and cognitive components are shown in Table 1.

### 3.2. Contemporaneous Network

**Figure 1:**
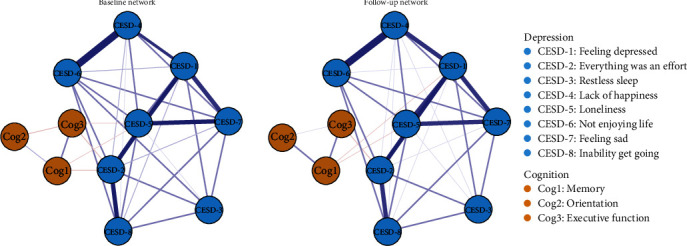
Contemporaneous networks of depression and cognitive components. *Note:* Contemporaneous networks for the baseline and follow-up datasets. The relationship of the components is indicated by the edge's color (blue = positive, red = negative), while the strength of the relationship is indicated by the edge's thickness (thicker = stronger).

Figure 1 shows two contemporaneous networks at (1) baseline and (2) follow-up assessments. In both networks, associations between nodes from the same symptom community were stronger than those based on the two distinct communities. Table S2 represents the undirected edges of the contemporaneous networks; associations within depression or cognition communities were positive, while associations between depression and cognition communities were negative. Of the undirected edges assessed across depression and cognition, the strongest edge was between “Everything was an effort” (CESD-2) and “Executive function” (Cog3; *r*_baseline_ = −0.15, *r*_follow-up_ = −0.19), followed by the edges between “Loneliness” (CESD-5) and “Memory” (Cog1; *r*_baseline_ = −0.09, *r*_follow-up_ = −0.06), and between “Loneliness” (CESD-5) and “Executive function” (Cog3; *r*_baseline_ = −0.07, *r*_follow-up_ = −0.05).

Bridge centrality of contemptuous networks is shown in Figure S1 and Table S4. “Lack of happiness” (CESD-4; BEI_baseline_ = −0.10, BEI_follow-up_ = −0.13) had the highest BEI value in the depression cluster. The two contemporaneous networks showed highly stability for centrality and edge weights, as both CS-coefficients were larger than 0.75 (Figure S4). The edge difference tests are shown in Figure S7. Additionally, edges weights of contemptuous networks were reliable (Figure S9).

### 3.3. Temporal Network

**Figure 2:**
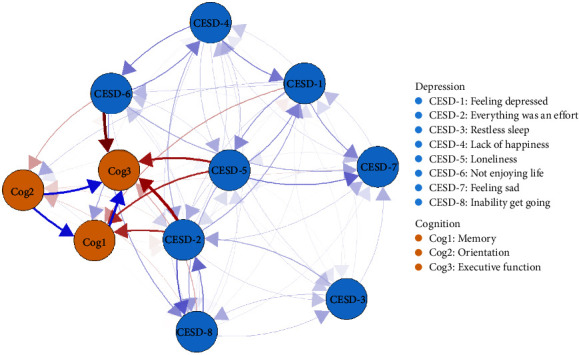
Temporal network of depressive and cognitive components. *Note:* The cross-lagged panel networks for depressive and cognitive components. The relationship of the components is indicated by the arrow's color (blue = positive, red = negative), and the strength of the relationship is indicated by the arrow's thickness (thicker = stronger). In these networks, autoregressive effects are excluded, and sex, age, married status, education level, drink, and smoke confounding were controlled.

All directed edges are shown in Figure S2. Autoregressive edges (NB: (mean effect = 0.29; Figure S3)) were significantly stronger than cross-lagged edges (mean effect = 0.015). To better understand the temporal associations between depression and cognition communities, we excluded autoregressive effects and included only cross-lagged associations in Figure 2. The arrows represented dynamic associations of each node within the depression and cognition temporal network model after controlling for all other neighboring nodes and covariates at baseline. The strongest directed edges that crossed clusters are listed in Table 2. “Not enjoying life” (CESD-6) at baseline was associated with lower “Executive function” (Cog3) at follow-up (*d* = −0.46), while higher “Everything was an effort” (CESD-2) at baseline was associated with lower “Executive function” (Cog3; *d* = −0.40) at follow-up. Moreover, higher “Loneliness” (CESD-5) at baseline was associated with lower “Executive function” (Cog3; *d* = −0.27) at follow-up. Higher “Loneliness” (CESD-5) and “Everything was an effort” (CESD-2) at baseline were associated with lower “Memory” (Cog1) at follow-up assessment (*d* = −0.22). The values of all edges across the two clusters are listed in Table S3.

**Figure 3:**
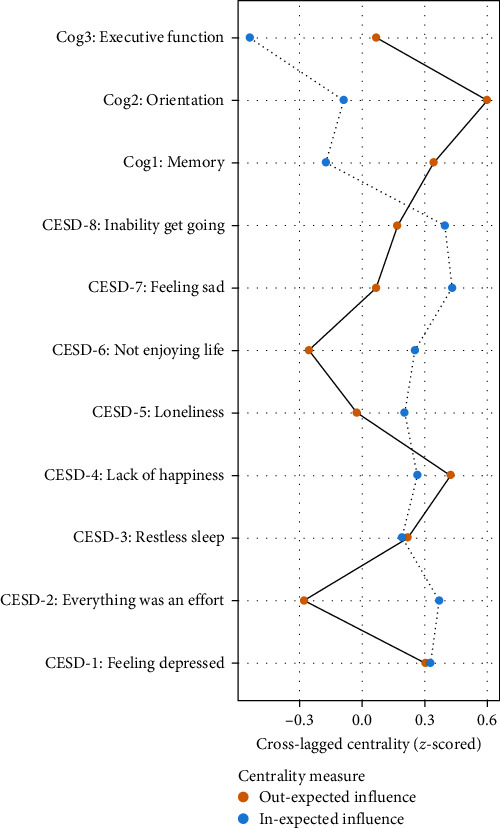
In-prediction and out-prediction of temporal network. *Note:* Symptom centrality estimates for the cross-lagged networks using *z* values. Greater values indicate greater centrality. Out-expected-influence is the degree to which individual symptom could predict other symptoms at the subsequent relevant point. In-expected-influence is the degree to which symptom is predicted by other symptoms at the subsequent relevant point.

Cross-lagged centrality estimates of the temporal network are shown in Figure 3 and Table S4, “Orientation” (Cog2) had the highest Out-EI value (Out-EI = 0.60), followed by “Lack of happiness” (CESD-4; Out-EI = 0.43). Analyses of In-EI values indicated that “Feeling sad” (CESD-7; In-EI = 0.43) and “Inability get going” (CESD-8; In-EI = 0.40) in the depression cluster had the highest values. Furthermore, edge, Out-EI, and In-EI values of the temporal network were stable, given that all CS-coefficients were larger than 0.5 (Figure S5). The centrality and edge difference tests (Figures S6 and S8) indicated centrality values and edge weights of each node within the temporal network were different from each other Also, the bootstrapped 95% CI of edge weights indicated the edge weights were relaible (Figure S10).

## 4. Discussion

To our knowledge, this is the first study to investigate the complex interplay between depressive symptoms and cognitive function components within a nationally representative sample of adults older than 50 years based on both cross-sectional and prospective network analyses, mapping unidirectional and directional relationships between depressive symptoms and cognitive components, respectively. The study found that baseline endorsements of the depressive symptom, “Not enjoying life” (CESD-6), predicted poorer executive function performance at follow-up. Baseline endorsements of “Everything was an effort” (CESD-2) and “Loneliness” (CESD-5) were also associated with poorer executive function or impaired memory at follow-up. These findings suggested that these depressive symptoms are viable potential targets for early intervention to prevent or alleviate cognitive decline in aging adults and further support and extend recent network evidence that more severe depressive symptoms predict future poorer cognitive function in older adults [20, 21].

The contemporaneous networks exhibited consistent patterns both at baseline and follow-up assessments, indicating that associations between depressive symptoms and cognitive functioning were stable and replicated within the same sample over time. Moreover, edges between depressive symptoms were stronger than those within cognitive components; this finding aligns with a prior depression and cognition network study conducted in Chinese older adults, wherein the top six strongest edges were from the depression community rather than the cognitive community [19]. These findings imply that nodes from the depression cluster are more likely to co-occur, supporting the assumption that depressive symptomatology is characterized by a range of related symptoms rather than isolated core symptoms [45]. Also, edge values within depression and cognition clusters were positive, indicating that increases in one symptom are related to potential increases in other symptoms from same cluster [46]. In contrast, edges across the depression and cognition clusters reflected strong negative associations, consistent with evidence from a longitudinal cohort study that also reported that more severe depressive symptoms were associated with poor cognitive function [47].

In the temporal network, all the strongest projections were from depressive symptoms to cognitive functions, indicating that depressive symptoms were more likely to predict subsequent cognitive impairments than the reverse. Similar patterns were also found in a prior CLPN study that reported no notable associations between cognitive functioning components and future depressive symptoms [20]. However, some researchers have found bidirectional relationships between depression and cognition [48]. For example, executive function may have a central influence on future psychiatric symptoms [49]. Moreover, in this study, the top three strongest predictive associations of depressive symptoms were directed toward executive function in line with the scar model theory premise [50–52] that elevations in depressed mood have more influence on reduced executive function over time than upon nonexecutive function cognitive abilities. Cohen's *d* effect sizes for associations of baseline “Not enjoying life,” “Everything was an effort,” and “Loneliness” endorsements with exacerbations in executive function deficits at the 2-year follow-up were small to medium (0.20–0.50) based on established guidelines [53] and were of sufficient strengths to be clinically meaningful in real-world circumstances. From a clinical and economic perspective, these findings provide empirical foundations for intervention studies targeting these depressive symptoms as potentially effective means of reducing specific cognitive declines in this at-risk population.

Executive function is characterized by a set of cognitive abilities for effective planning, problem solving, reasoning, and communicating [54]. “Not enjoying life” (CESD-6) and “Loneliness” (CESD-5) predicted executive function decrements among older adults, suggesting that individuals who do not have pleasurable or rewarding activities or have less meaningful social contact than they desire are at risk for experiencing executive function declines at subsequent time points. Such effects may be a partial function of reduced engagement in physical and social activities [55, 56]. A large-scale cohort study conducted over 6 years found that older adults who engaged in higher levels of physical activity tended to maintain higher levels of executive function [48]. Moreover, a previous study found that individuals who participated in short-term social interactions, such as getting to know others and playing brain games, experienced subsequent improvements in processing speed and executive function [57]. A meta-analysis reported that regular physical exercise can effectively improve global cognition and executive function in older adults with mild cognitive impairment [58]. In tandem with these findings, our results suggest that interventions aimed at increasing engagement in physical and social activities to enhance the enjoyment of life and/or reduce loneliness could be an important factor in improving executive function. However, the types and levels of exercise or social activity that can alleviate declines in executive function are uncertain and warrant further study.

“Everything was an effort” (CESD-2) reflects a perceived burden and lack of motivation in performing even daily tasks (Bech, 2012) and was also associated with future declines in executive function. Executive functions are complex and their performance depends on one's willingness to invest effort and closely tied to one's motivation [59]. Previous research has found that motivation and executive function overlap with each other because they share common brain regions [60]. Recent research has also shown strategies to increase motivation such as by providing positive feedback, providing incentives, and encouraging social support that hold promise as means of promoting better executive function performance among older adults [61, 62].

“Everything was an effort” (CESD-2) and “Loneliness” (CESD-5) could also substantially influence memory of older adults in our sample. Memory decline is common among the elderly and a key symptom of cognitive impairment that interferes with other cognitive domains [60]. Memory involves complex mechanisms, stages, and types. For instance, immediate memory can be transformed into delayed memories through encoding and rehearsal procedures that allow short-term information to be stored in a long-term form [63]. Interventions aimed at replacing a sense of futility and loneliness with increased engagement may help to improve individuals' cognitive performance, particularly memory. Previous research found that engagement of social or intellectual activities can enhance cognitive reserves in the brain, thereby preventing age-related cognitive decline [11]. From an individual perspective, engaging in in-person activities or maintaining daily contact with friends are useful ways to provide companionship and life engagement for older adults [64]. From the perspective of policymaking, increases in community resources to support regular social activities and social support systems are important avenues for reducing losses of enjoyment and alleviating loneliness experienced by older adults [65] and consequently mitigating the personal and economic costs of cognitive deterioration among older adults in society [66].

BEI refers to the overall connectivity of each node with its neighboring nodes at the same time point [35], while Out-EI represents the connectivity of each node with other nodes at the subsequent measurement occasion [18]. In our network results, the depression symptom with a high BEI value and Out-EI value was “Lack of happiness” (CESD-4), which was somewhat expected given that anhedonia is considered to be a fundamental symptom of depression [67–69]. In addition, happiness has been linked to lower risk of all-cause dementia in older adults [70]. A prior study conducted on older adults found that happier individuals at baseline perform better in visuospatial memory and processing speed (a type of executive function) tasks compared to less happy individuals [71]. Additionally, happiness has been linked to momentary attention and cognitive flexibility [72]. Moreover, dopaminergic theory posits that positive experiences could elevate dopamine levels in specific brain regions such as the prefrontal cortex [73]. This may be one pathway by which happiness enhances cognitive functioning. Therefore, increasing feelings of happiness and positive effect in older adults would be useful to protect or slow down the activation of depression–cognition connection. In-EI indicates the extent to which each node is predicted by all other nodes at the subsequent relevant point [18]. We found that “Feeling sad” (CESD-7) had the highest In-EI index; this result may be because changes in other depressive symptoms often predict changes in “Feeling sad,” which is a core symptom of depression [74].

This study has several strengths. First, use of a large nationally representative cohort of over 6,000 participants provided sufficient power to detect the true internal associations of depression and cognition that apply to older adults in general. Second, we used both cross-sectional and longitudinal network approaches to model unidirectional and directional associations between nodes; this two-pronged strategy enabled us not only to map a static network structure of depression and cognition at symptoms levels but also to capture dynamic changes between depression and cognition over time. Last, we controlled for potentially confounding factors that may influence associations of depression with cognition (e.g., sex, age, married status, education level, drink, and smoke status).

Despite its strengths, this study had several shortcomings. First, participants who did not complete the baseline assessment of depressive symptoms and cognitive function were excluded from analyses. This may have resulted in an attrition bias by which the final sample was not entirely representative of the initial sample. Second, executive function is a multifaceted concept that encompasses various cognitive abilities, including verbal fluency, working memory, processing speed, and attention [75]. Due to data limitations in the ELSA, only verbal fluency test results were included to represent the executive function domain following previous published ELSA studies [70, 76]. Third, the network models were based on two time points with a 2-year interval between them prior to the COVID-19 pandemic. Future network studies could incorporate a more prolonged time period to explore interrelationships of depression with cognition over extended periods following the pandemic. Last, data were collected in England, which may limit the generalizability of results to other cultural contexts.

In conclusion, findings of this network analysis study highlighted the dynamic and predictive relationships between particular depressive symptoms and specific cognitive declines in older adults. Findings provide foundations for tests of interventions targeting key depressive symptoms (“Not enjoying life” (CESD-6), “Everything was an effort” (CESD-2), and “Loneliness” (CESD-5)) that have the potential to alleviate future declines in executive function or memory abilities. Furthermore, strategies aimed at improving happiness could be useful in reducing the co-occurrence of depression and cognitive decline in this population.

## Figures and Tables

**Table 1:** Node labels and item descriptions.

Labels	Nodes	Survey time	
		Baseline survey	Follow-up survey
Depression		*N* (%)	*N* (%)
Feeling depressed	CESD-1	678 (10.5)	649 (10.4)
Everything was an effort	CESD-2	1,126 (17.5)	1,183 (18.3)
Restless sleep	CESD-3	2,327 (36.1)	2,756 (42.8)
Lack of happiness	CESD-4	531 (8.2)	542 (8.4)
Loneliness	CESD-5	667 (10.3)	652 (10.1)
Not enjoying life	CESD-6	474 (7.3)	518 (8.0)
Feeling sad	CESD-7	1,154 (17.9)	1,104 (17.1)
Inability get going	CESD-8	1,100 (17.1)	1,196 (18.6)

**Cognition**		**Mean (SD)**	**Mean (SD)**

Memory	Cog1	11.04 (3.541)	10.62 (3.665)
Orientation	Cog2	3.80 (0.496)	3.7 (0.593)
Executive function	Cog3	—	—

*Note*: Executive function as transformed a categorized variables without mean values.

Abbreviations: CES-D, Center for Epidemiological Studies–Depression Scale; Cog, cognitive components.

**Table 2:** Strongest directed cross-lagged edges of temporal network.

Rank	Node-out	Node-in	Cohen's *d*
1	CESD-6: Not enjoying life	Cog3: Executive function	−0.46
2	CESD-2: Everything was an effort	Cog3: Executive function	−0.40
3	CESD-5: Loneliness	Cog3: Executive function	−0.27
4	CESD-5: Loneliness	Cog1: Memory	−0.22
5	CESD-2: Everything was an effort	Cog1: Memory	−0.22

## Data Availability

The data can be assessed from https://www.elsa-project.ac.uk/.

## Supporting Information

Supplementary Materials Table S1: Demographic characteristics for each sampling time (*N* = 6,433). Table S2: Weighted adjacency matrix of the contemporaneous network model. Table S3: Cross-lagged edges of temporal network. Table S4: Centrality and predictability values. Figure S1: Centrality of Contemporaneous networks. Figure S2: The cross-lagged panel networks (including autoregression edges). Figure S3: Autoregressive edges for each symptom in each network. Figure S4: Bootstrapped Stability of contemporaneous network indices. Figure S5: Bootstrapped stability of temporal network indices. Figure S6: Bootstrapped significance of differences of out-expected-influence and in-expected-influence in temporal network. Figure S7: Bootstrapped significance of edge differences in contemporaneous network. Figure S8: Bootstrapped significance of edge differences in temporal network. Figure S9: Bootstrapped 95% confidence intervals around contemporaneous network edges. Figure S10: Bootstrapped 95% confidence intervals around temporal network edges.
