# Supplementary material for: Depressive Symptoms and Cognitive Function in Older Adults: A Cross-Lagged Network Analysis
Source: Depress Anxiety. 2024 Sep 21;2024:6166775. doi: 10.1155/2024/6166775 (PMC11919221; doi:10.1155/2024/6166775)
Supplement: Supplementary Materials — Table S1: Demographic characteristics for each sampling time (N = 6,433). Table S2: Weighted adjacency matrix of the contemporaneous network model. Table S3: Cross-lagged edges of temporal network. Table S4: Centrality and predictability values. Figure S1: Centrality of Contemporaneous networks. Figure S2: The cross-lagged panel networks (including autoregression edges). Figure S3: Autoregressive edges for each symptom in each network. Figure S4: Bootstrapped Stability of contemporaneous network indices. Figure S5: Bootstrapped stability of temporal network indices. Figure S6: Bootstrapped significance of differences of out-expected-influence and in-expected-influence in temporal network. Figure S7: Bootstrapped significance of edge differences in contemporaneous network. Figure S8: Bootstrapped significance of edge differences in temporal network. Figure S9: Bootstrapped 95% confidence intervals around contemporaneous network edges. Figure S10: Bootstrapped 95% confidence intervals around temporal network edges. [file 6166775.f1.docx]

**Depressive symptoms and cognitive function in older adults: a cross lagged network analysis**

**Supplementary materials**

**1. Supplement methods**

**2. Supplement tables and figures**

Table S1. Demographic characteristics for each sampling time (N=6,433)

Table S2. Weighted adjacency matrix of the contemporaneous network model

Table S3. Cross lagged edges of temporal network

Table S4. Centrality and predictability values

Figure S1. Centrality of Contemporaneous networks

Figure S2: The cross-lagged panel networks (including auto-regression edges)

Figure S3: Autoregressive edges for each symptom in each network

Figure S4: Bootstrapped Stability of contemporaneous network indices

Figure S5: Bootstrapped Stability of temporal network indices

Figure S6: Bootstrapped significance of differences of Out-expected-influence and In-expected-influence in temporal network

Figure S7: Bootstrapped significance of edge differences in contemporaneous network

Figure S8: Bootstrapped significance of edge differences in temporal network

Figure S9: Bootstrapped 95% confidence intervals around contemporaneous network edges

Figure S10: Bootstrapped 95% confidence intervals around temporal network edges

**1. Supplement methods**

**Fluency recoded for executive function index**

Value = 0.0 Label = 0 animals

Value = 1.0 Label = 1-7 animals

Value = 2.0 Label = 8-12 animals

Value = 3.0 Label = 13-15 animals

Value = 4.0 Label = 16-17 animals

Value = 5.0 Label = 18-19 animals

Value = 6.0 Label = 20-21 animals

Value = 7.0 Label = 22-24 animals

Value = 8.0 Label = 25-29 animals

Value = 9.0 Label = 30+ animals

**2. Supplement tables and figures**

**Table S1. Demographic characteristics at baseline (N=6,433)**

| **Variables** | **N** | **%** |
| --- | --- | --- |
| Male | 2,800 | 43.5 |
| Married | 4,214 | 65.5 |
| Educated | 5,135 | 79.8 |
| Current smoke | 964 | 14.9 |
| Current alcoholic drink | 4,521 | 70.3 |
|  | **Mean** | **SD** |
| Age (years) | 68.47 | 8.95 |
| CESD-8 total | 2.93 | 1.29 |
| Notes: CESD-8: Eight items of Center for Epidemiological Studies Depression Scale | | |

**Table S2. Weighted adjacency matrix of the contemporaneous network model**

| **Baseline** | | | | | | | | | | | |
| --- | --- | --- | --- | --- | --- | --- | --- | --- | --- | --- | --- |
|  | CESD1 | CESD2 | CESD3 | CESD4 | CESD5 | CESD6 | CESD7 | CESD8 | Cog1 | Cog2 | Cog3 |
| CESD1 |  |  |  |  |  |  |  |  |  |  |  |
| CESD2 | 0.80 |  |  |  |  |  |  |  |  |  |  |
| CESD3 | 0.28 | 0.26 |  |  |  |  |  |  |  |  |  |
| CESD4 | 0.59 | 0.15 | 0.00 |  |  |  |  |  |  |  |  |
| CESD5 | 0.42 | 0.00 | 0.12 | 0.32 |  |  |  |  |  |  |  |
| CESD6 | 0.21 | 0.34 | 0.22 | 1.38 | 0.31 |  |  |  |  |  |  |
| CESD7 | 0.87 | 0.17 | 0.21 | 0.38 | 0.81 | 0.26 |  |  |  |  |  |
| CESD8 | 0.18 | 1.09 | 0.31 | 0.08 | 0.24 | 0.21 | 0.25 |  |  |  |  |
| Cog1 | 0.00 | -0.15 | 0.00 | 0.00 | -0.09 | 0.00 | 0.00 | -0.04 |  |  |  |
| Cog2 | 0.00 | 0.00 | 0.00 | 0.00 | 0.00 | 0.00 | 0.00 | 0.00 | 0.15 |  |  |
| Cog3 | 0.00 | -0.15 | 0.00 | 0.00 | -0.07 | 0.00 | 0.00 | 0.00 | 0.40 | 0.08 | 0.00 |
| **Follow-up** | | | | | | | | | | | |
|  | CESD1 | CESD2 | CESD3 | CESD4 | CESD5 | CESD6 | CESD7 | CESD8 | Cog1 | Cog2 | Cog3 |
| CESD1 |  |  |  |  |  |  |  |  |  |  |  |
| CESD2 | 0.77 |  |  |  |  |  |  |  |  |  |  |
| CESD3 | 0.20 | 0.35 |  |  |  |  |  |  |  |  |  |
| CESD4 | 0.66 | 0.11 | 0.10 |  |  |  |  |  |  |  |  |
| CESD5 | 0.50 | 0.09 | 0.11 | 0.26 |  |  |  |  |  |  |  |
| CESD6 | 0.13 | 0.42 | 0.00 | 1.37 | 0.23 |  |  |  |  |  |  |
| CESD7 | 0.83 | 0.12 | 0.31 | 0.38 | 0.68 | 0.23 |  |  |  |  |  |
| CESD8 | 0.14 | 1.01 | 0.30 | 0.07 | 0.21 | 0.39 | 0.25 |  |  |  |  |
| Cog1 | -0.10 | -0.04 | 0.00 | 0.00 | -0.06 | 0.00 | 0.00 | -0.07 |  |  |  |
| Cog2 | 0.00 | 0.00 | 0.00 | 0.00 | 0.00 | 0.00 | 0.00 | -0.05 | 0.20 |  |  |
| Cog3 | 0.00 | -0.19 | 0.00 | 0.00 | -0.05 | 0.00 | 0.00 | 0.00 | 0.45 | 0.12 | 0.00 |

**Table S3. Cross lagged edges of temporal network**

| Node Out | Node In | value |
| --- | --- | --- |
| CESD6: Not enjoying life | Cog3: Executive function | -0.46 |
| CESD2: Everything was an effort | Cog3: Executive function | -0.40 |
| CESD5: Loneliness | Cog3: Executive function | -0.27 |
| CESD5: Loneliness | Cog1: Memory | -0.23 |
| CESD2: Everything was an effort | Cog1: Memory | -0.22 |
| CESD1: Feeling depressed | Cog1: Memory | -0.08 |
| CESD6: Not enjoying life | Cog2: Orientation | -0.07 |
| CESD8: Inability get going | Cog3: Executive function | -0.07 |
| CESD8: Inability get going | Cog2: Orientation | -0.06 |
| CESD2: Everything was an effort | Cog2: Orientation | -0.03 |
| CESD7: Feeling sad | Cog2: Orientation | -0.03 |
| CESD4: Lack of happiness | Cog1: Memory | -0.01 |
| Cog2: Orientation | CESD2: Everything was an effort | -0.01 |
| Cog2: Orientation | CESD1: Feeling depressed | -0.01 |
| Cog1: Memory | CESD2: Everything was an effort | -0.01 |
| Cog2: Orientation | CESD6: Not enjoying life | -0.01 |
| Cog1: Memory | CESD8: Inability get going | 0.00 |
| Cog1: Memory | CESD1: Feeling depressed | 0.00 |
| Cog3: Executive function | CESD2: Everything was an effort | 0.00 |
| Cog2: Orientation | CESD8: Inability get going | 0.00 |
| Cog2: Orientation | CESD5: Loneliness | 0.00 |
| Cog1: Memory | CESD4: Lack of happiness | 0.00 |
| Cog1: Memory | CESD5: Loneliness | 0.00 |
| Cog3: Executive function | CESD5: Loneliness | 0.00 |
| Cog3: Executive function | CESD1: Feeling depressed | 0.00 |
| Cog3: Executive function | CESD8: Inability get going | 0.00 |
| Cog3: Executive function | CESD4: Lack of happiness | 0.00 |
| Cog3: Executive function | CESD6: Not enjoying life | 0.00 |
| Cog1: Memory | CESD3: Restless sleep | 0.00 |
| Cog2: Orientation | CESD3: Restless sleep | 0.00 |
| Cog3: Executive function | CESD3: Restless sleep | 0.00 |
| Cog1: Memory | CESD6: Not enjoying life | 0.00 |
| Cog1: Memory | CESD7: Feeling sad | 0.00 |
| Cog2: Orientation | CESD7: Feeling sad | 0.00 |
| Cog3: Executive function | CESD7: Feeling sad | 0.00 |
| CESD3: Restless sleep | Cog1: Memory | 0.00 |
| CESD6: Not enjoying life | Cog1: Memory | 0.00 |
| CESD7: Feeling sad | Cog1: Memory | 0.00 |
| CESD8: Inability get going | Cog1: Memory | 0.00 |
| CESD3: Restless sleep | Cog2: Orientation | 0.00 |
| CESD4: Lack of happiness | Cog2: Orientation | 0.00 |
| CESD1: Feeling depressed | Cog3: Executive function | 0.00 |
| CESD3: Restless sleep | Cog3: Executive function | 0.00 |
| CESD4: Lack of happiness | Cog3: Executive function | 0.00 |
| CESD7: Feeling sad | Cog3: Executive function | 0.00 |
| Cog2: Orientation | CESD4: Lack of happiness | 0.00 |
| CESD5: Loneliness | Cog2: Orientation | 0.01 |
| CESD1: Feeling depressed | Cog2: Orientation | 0.06 |

**Table S4. Components EI and predictability values**

|  | **Baseline (2016-2017)** | | **Follow-up (2018-2019)** | | **Baseline to follow up** | |
| --- | --- | --- | --- | --- | --- | --- |
| **Nodes** | **BEI** | **Predictability** | **BEI** | **Predictability** | **Out EI** | **In EI** |
| CESD1: Feeling depressed | -0.31 | 0.894 | -0.42 | 0.817 | 0.31 | 0.33 |
| CESD2: Everything was an effort | -0.48 | 0.825 | -0.58 | 0.571 | -0.28 | 0.37 |
| CESD3: Restless sleep | -0.11 | 0.639 | -0.15 | 0.916 | 0.22 | 0.19 |
| CESD4: Lack of happiness | -0.10 | 0.918 | -0.13 | 0.899 | 0.43 | 0.27 |
| CESD5: Loneliness | -0.25 | 0.896 | -0.28 | 0.919 | -0.02 | 0.20 |
| CESD6: Not enjoying life | -0.16 | 0.926 | -0.18 | 0.828 | -0.25 | 0.25 |
| CESD7: Feeling sad | -0.19 | 0.82 | -0.22 | 0.814 | 0.07 | 0.43 |
| CESD8: Inability get going | -0.41 | 0.829 | -0.46 | 0.308 | 0.17 | 0.40 |
| Cog1: Memory | -1.06 | 0.233 | -1.15 | 0.121 | 0.35 | -0.17 |
| Cog2: Orientation | -0.06 | 0.060 | -0.25 | 0.29 | 0.60 | -0.09 |
| Cog3: Executive function | -0.90 | 0.218 | -1.02 | 0.817 | 0.07 | -0.54 |
| Note: EI: Expected Influence; BEI: Bridge Expected Influence; Predictability of depression based on CCmarg, Predictability of cognition based on R2. BEI, Out-EI and In- EI are based on z score value. | | | | | | |

**Figure S1. Centrality of Contemporaneous networks**

**
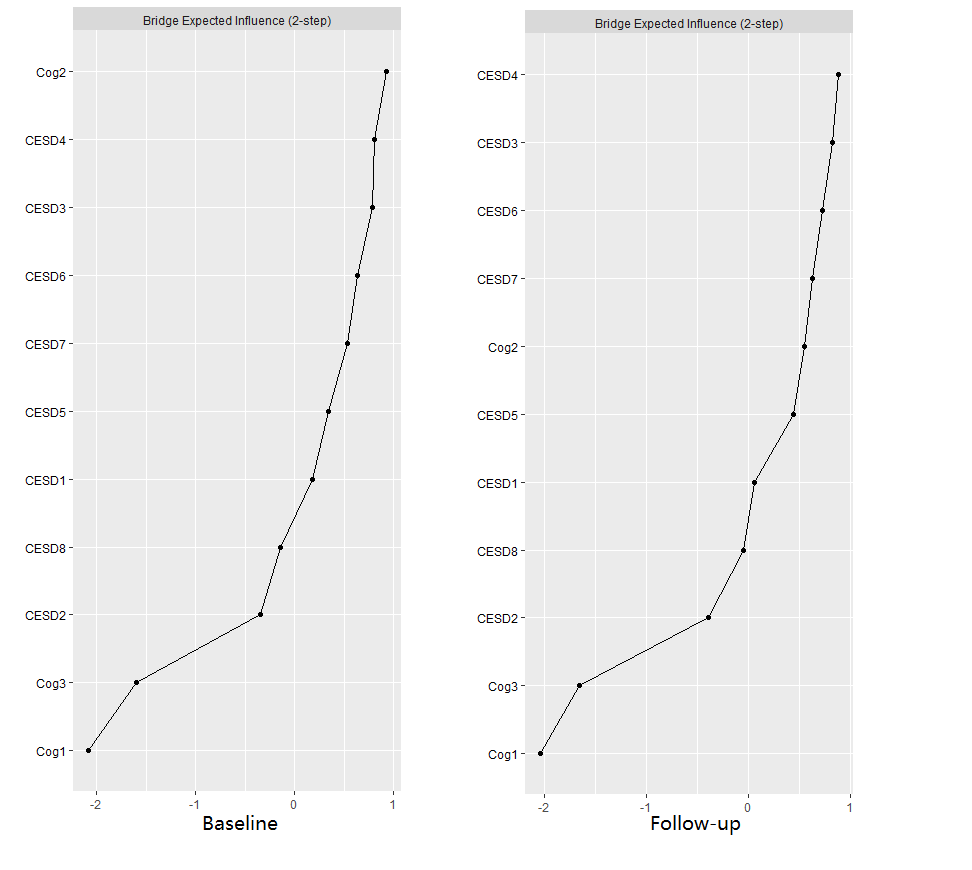
**

**Figure S2: The cross-lagged panel networks (including auto-regression edges)**

**
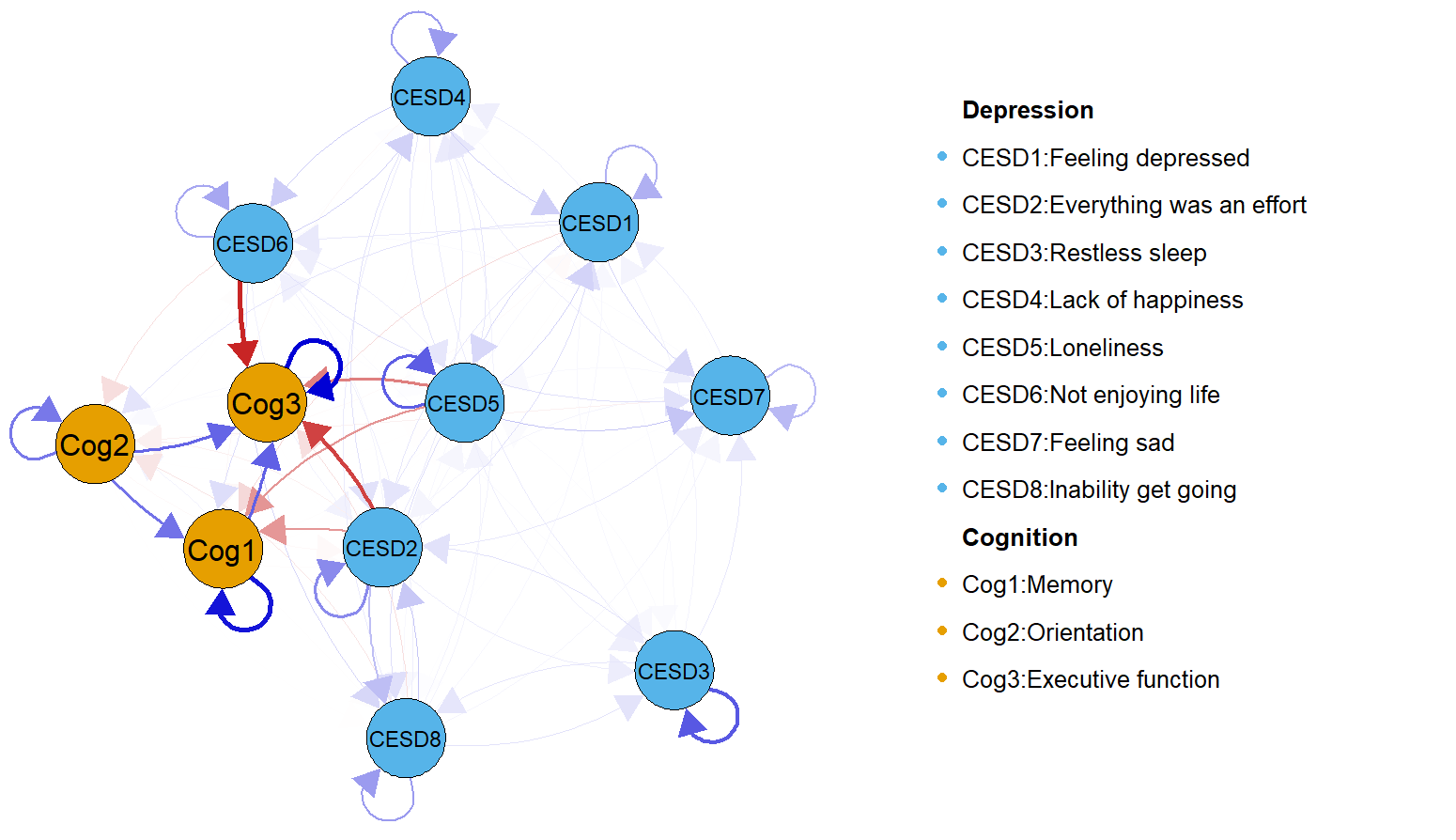
**

**Figure S3: Autoregressive edges for each symptom in temporal network
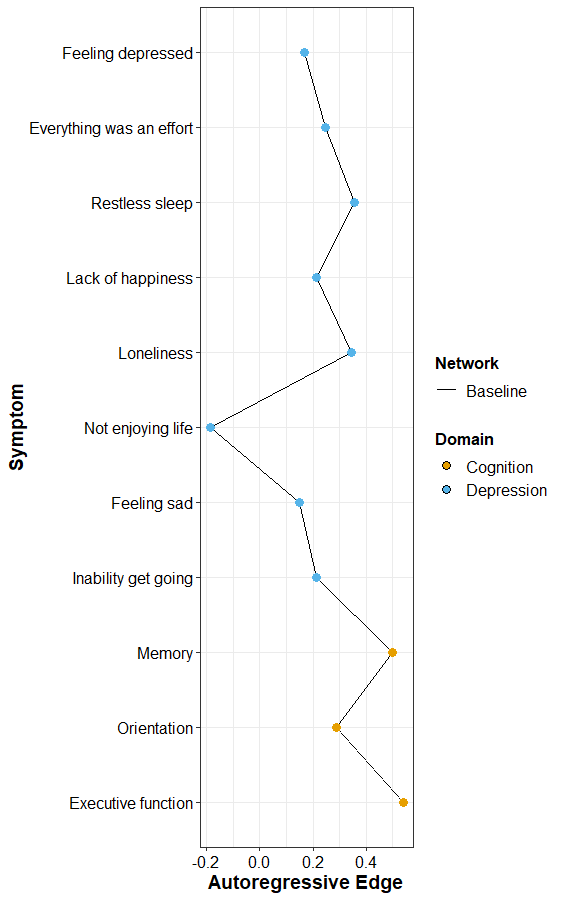
**

**Figure S4: Bootstrapped Stability of contemporaneous network indices**

**
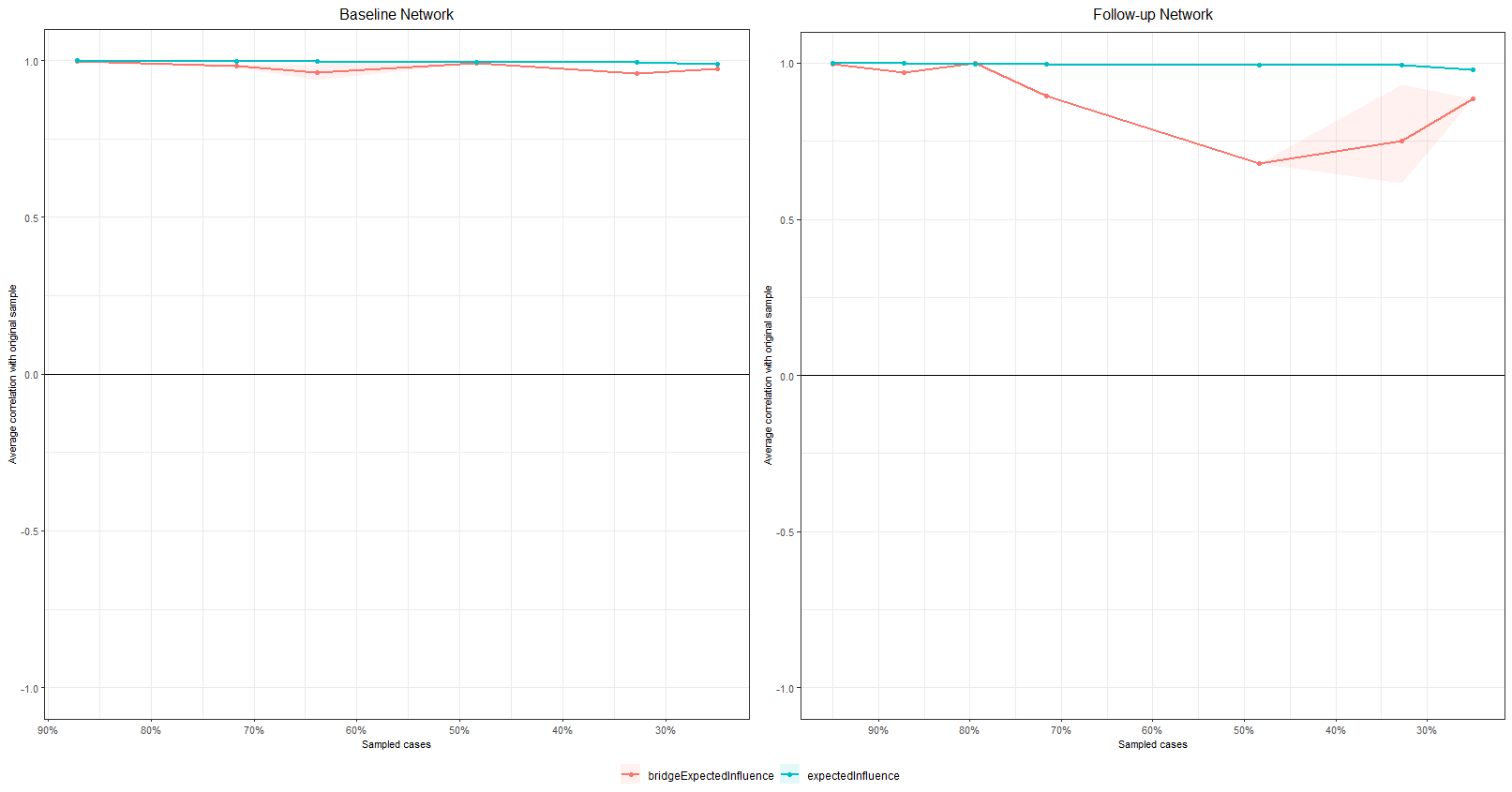
**

**Figure S5: Bootstrapped Stability of temporal network indices**

**
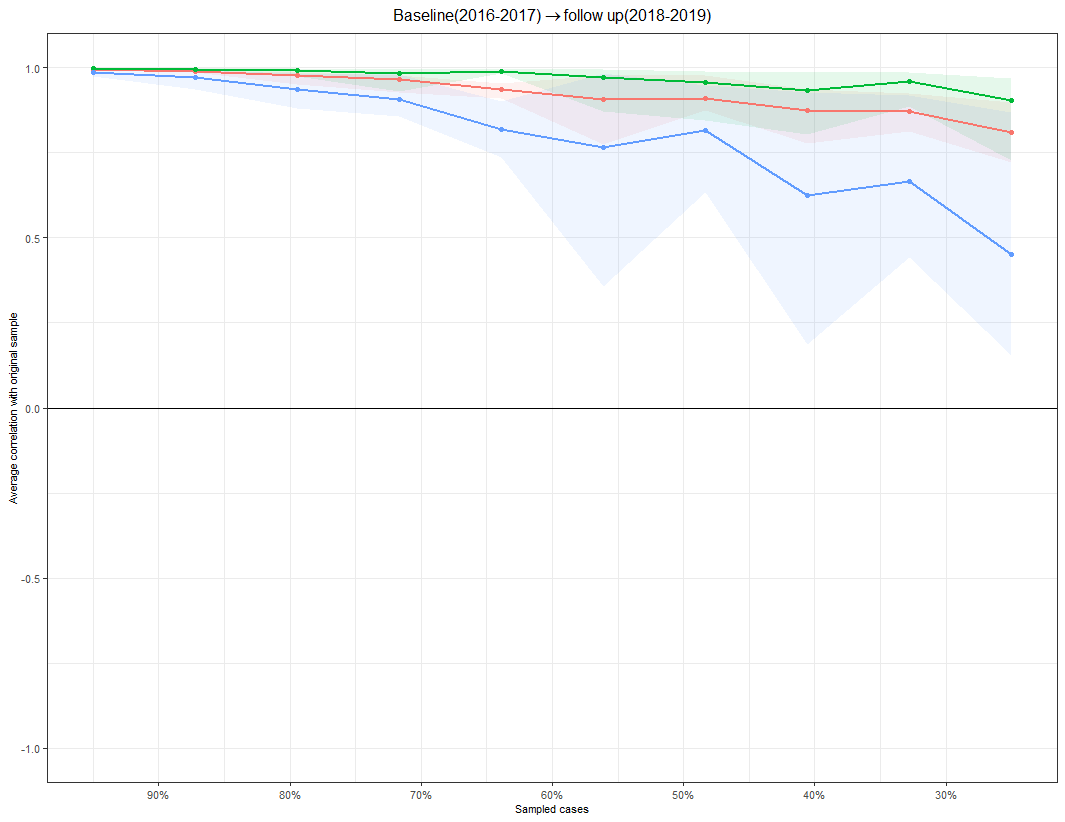
**

Note: the x-axis represents the % of the original sample used to calculate the centrality. The green indicated the edge influence, red line indicated the in-Expected Influence, the blue line indicated the out-Expected Influence

edge: 0.75 (CS-coefficient is highest level tested)

- For more accuracy, run bootnet(..., caseMin = 0.672, caseMax = 1)

inExpectedInfluence: 0.75 (CS-coefficient is highest level tested)

- For more accuracy, run bootnet(..., caseMin = 0.672, caseMax = 1)

outExpectedInfluence: 0.517

- For more accuracy, run bootnet(..., caseMin = 0.439, caseMax = 0.59

**Figure S6: Bootstrapped significance of differences of** **Out-expected-influence and In-expected-influence in temporal network**

**
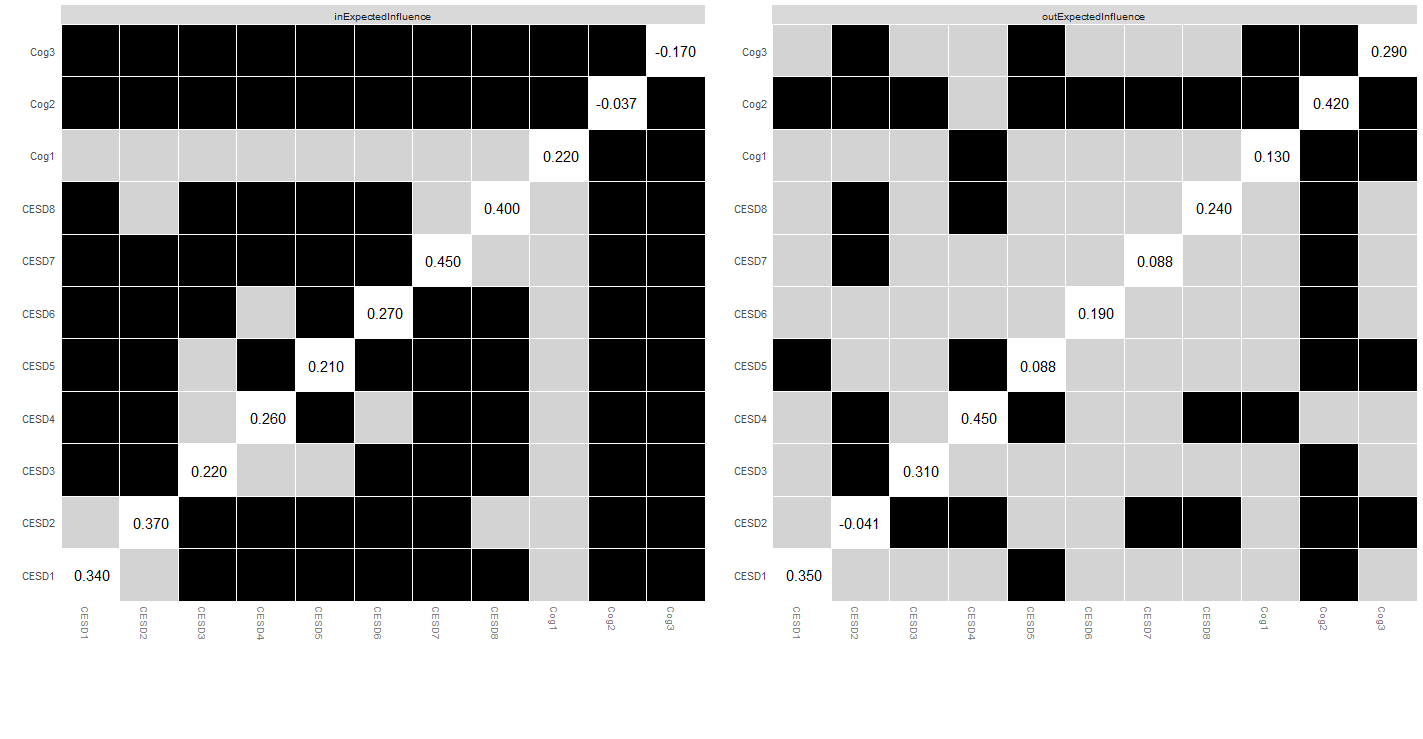
**

Note: The left panel was In-expected influence, the right panel was Out-expected Influence. Grey boxes indicate nodes that do not differ significantly from one-another (p <.05) and black boxes represent nodes that do differ significantly from one-another. White boxes in the centrality plot show the value of Outexpected-influence and Inexpected-influence node.

**Figure S7: Bootstrapped significance of edge differences in contemporaneous network**

**
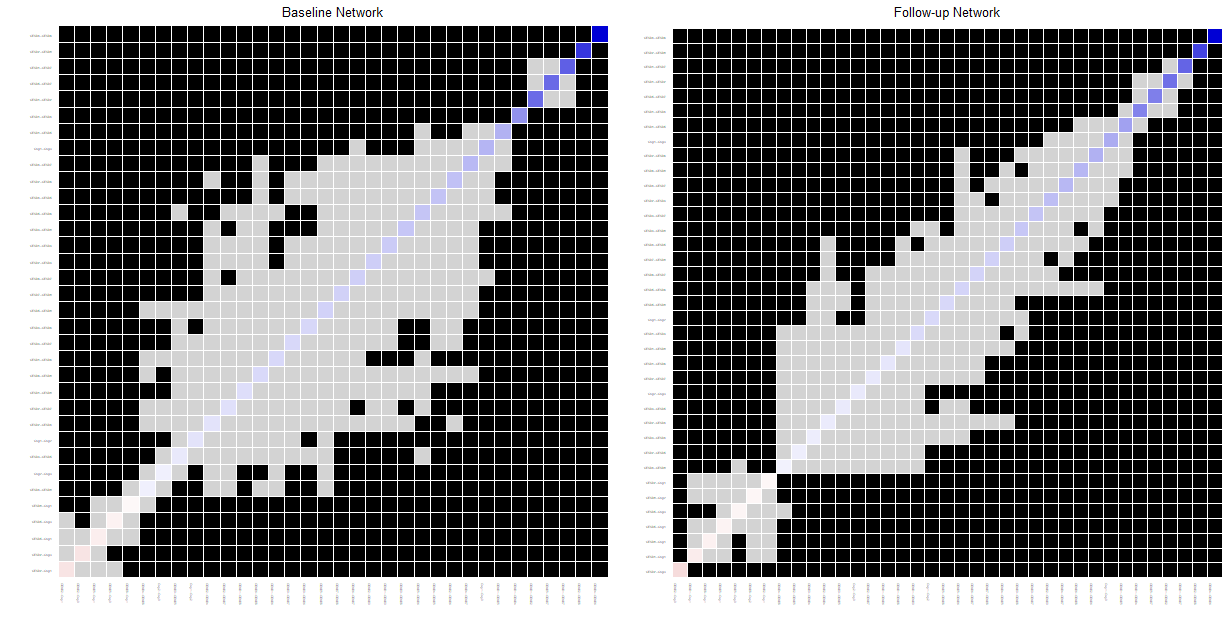
**

**Figure S8: Bootstrapped significance of edge differences in temporal network**


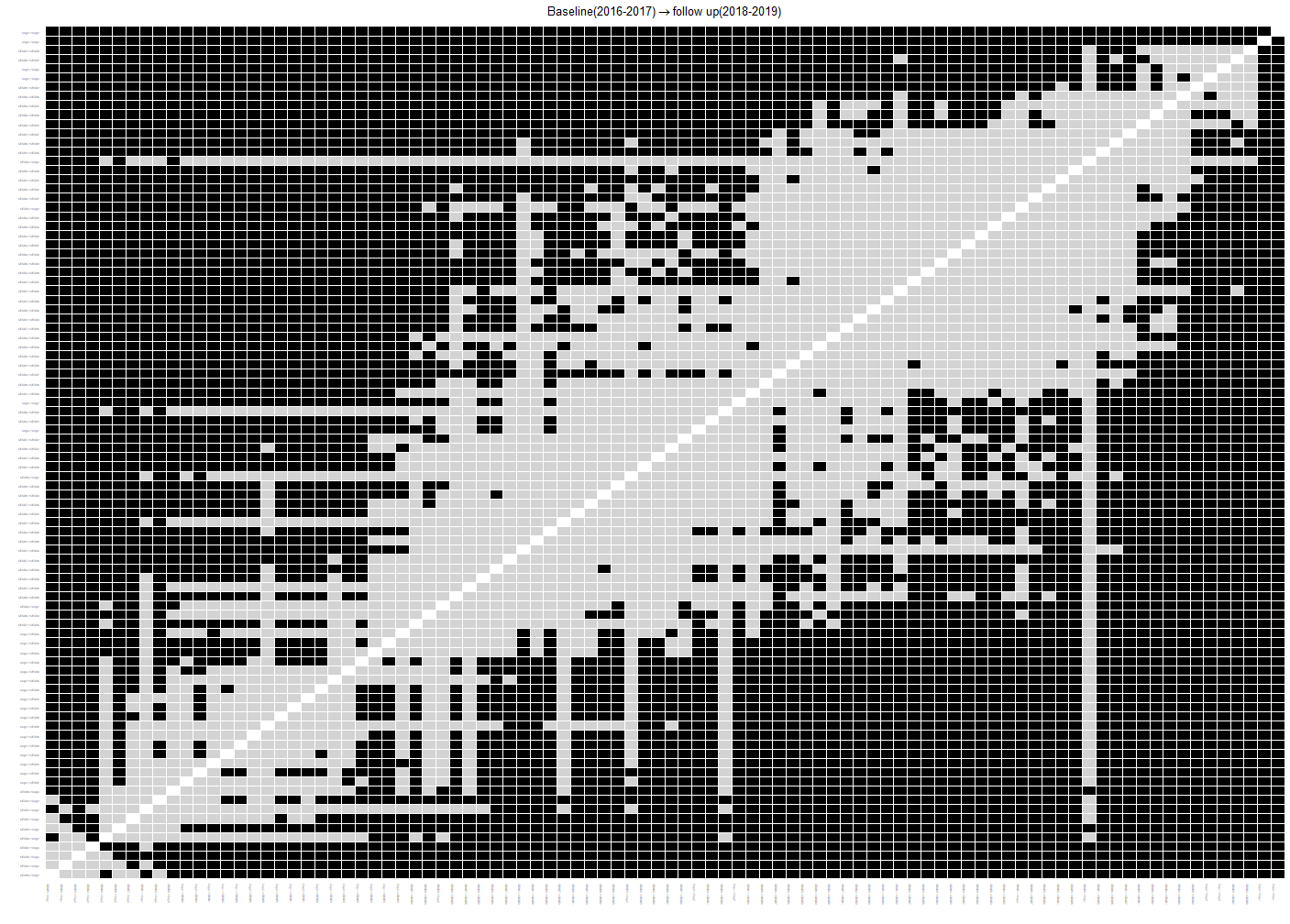


Note: Grey boxes indicate edges that do not differ significantly from one-another (p <.05) and black boxes represent edges that do differ significantly from one-another.

**Figure S9: Bootstrapped 95% confidence intervals around contemporaneous network edges**


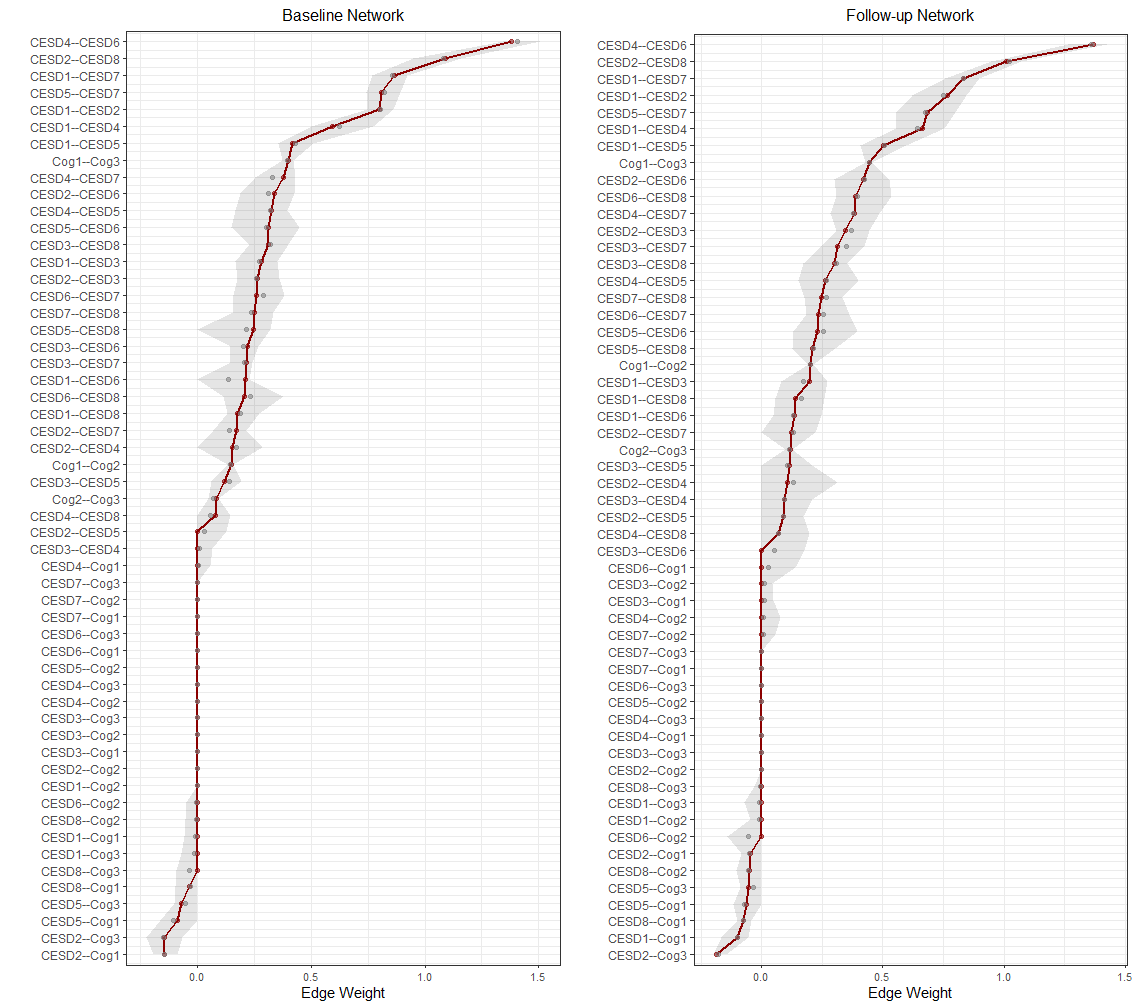


**Figure S10: Bootstrapped 95% confidence intervals around temporal network edges**


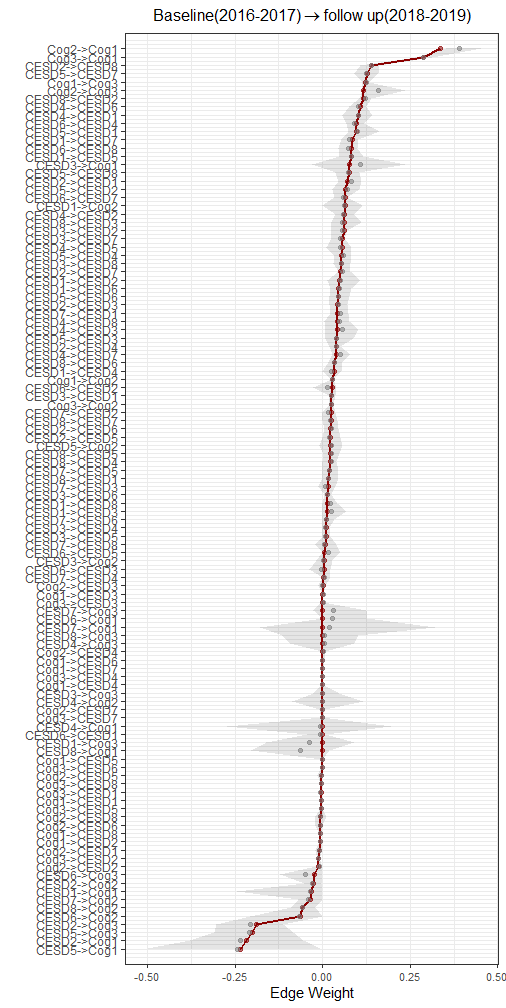


Notes: The plot shows the sample interrelations with 1000 bootstrap iterations (i.e., edge weights; red dots), the means of the bootstrapped interrelations (i.e., edge weights; black dots), and the bootstrap confidence intervals.
